# Persecutory ideation and insomnia: Findings from the second British National Survey Of Psychiatric Morbidity

**DOI:** 10.1016/j.jpsychires.2010.03.018

**Published:** 2010-11

**Authors:** Daniel Freeman, Traolach Brugha, Howard Meltzer, Rachel Jenkins, Daniel Stahl, Paul Bebbington

**Affiliations:** a King’s College London, Department of Psychology, PO Box 077, Institute of Psychiatry, Denmark Hill, London SE5 8AF, UK; b Department of Health Sciences, University of Leicester, UK; c King's College London, Health Service and Population Research, Institute of Psychiatry, UK; d King's College London, Department of Biostatistics, Institute of Psychiatry, UK; e Department of Mental Health Sciences, University College London, UK

**Keywords:** Delusions, Paranoia, Insomnia, Anxiety, Worry

## Abstract

It is clinically and theoretically plausible that insomnia contributes to the development and maintenance of paranoid fears. The primary aim of the study was to establish in a large sample whether insomnia and paranoia are associated more strongly than by chance. Cross-sectional data on paranoia, insomnia, anxiety, worry, depression, irritability, and cannabis use were obtained from the second British National Survey of Psychiatric Morbidity, a general population survey of adults aged 16–74 years living in Great Britain (*N* = 8580). It was found that insomnia was associated with an approximately two to threefold increase in paranoid thinking. Paranoia and insomnia were both strongly associated with the presence of anxiety, worry, depression, irritability and cannabis use. In a path analysis the association of paranoia and insomnia was partially explained by the affective symptoms, and, to a much lesser degree, cannabis use. The results are consistent with recent developments in the cognitive understanding of persecutory delusions, in which insomnia, negative affect, and substance use are identified as key factors. Longitudinal studies of insomnia and paranoia, and tests of the effects of sleep interventions on levels of paranoia, are now required to examine causality.

## 1 Introduction

Difficulties in falling or staying asleep are common. Approximately 30% of the general population experience current symptoms of insomnia, with a third of this group having chronic insomnia (Ohayon, 2002; Walsh, 2004; Morin et al., 2006; Stewart et al., 2006). It is only in the last few years that it has been recognised that paranoid thinking is almost as prevalent. At least 25% of the general public regularly experience paranoid thoughts, and the lifetime prevalence of persecutory delusions is approximately 5% (Freeman and Freeman, 2008; Rutten et al., 2008). Intriguingly, both insomnia and paranoia have been linked with negative affect. Sleep difficulties are a risk factor for developing emotional disorders such as anxiety and depression (Ford and Kamerow, 1989; Breslau et al., 1996; Morphy et al., 2007), while negative affective states predict the occurrence of paranoid thinking (see Freeman, 2007; Bentall et al., 2008). Therefore insomnia exacerbates factors shown in separate research to predict paranoia. It is plausible that sleep difficulties contribute to the occurrence of paranoia. An unfortunate direct example of this can be seen in a treatment case series where sleep deprivation for 36 h led to a worsening of delusions in individuals with depressive psychosis (Benedetti et al., 1999).

Sleep difficulties have been noted in people with schizophrenia, for example as a common prodromal symptom (Birchwood et al., 1989; Yung and McGorry, 1996), but only one study has directly examined insomnia and paranoia. In 300 individuals from the general population, insomnia and paranoid ideation were strongly associated, and the association was partly explained by the presence of anxiety and depression (Freeman et al., 2009). In a small group of patients with persecutory delusions (and a diagnosis of schizophrenia), moderate or severe insomnia was present in more than 50%. This provides preliminary evidence of the potential importance of insomnia for understanding paranoia. The central aim of the current study is to corroborate this association by examining data from the British National Survey of Psychiatric Morbidity (2000) (Singleton et al., 2001a), a large population-based survey with random sampling of community members. The key prediction is that insomnia is associated with paranoia. A secondary prediction is that the association will be partly explained by levels of negative affect and associated experiences (anxiety, worry, depression, and irritability). When attempting to account for the potential association it is relevant to note that substance use has been separately linked to sleep difficulties (e.g. Roth et al., 2006; Stein et al., 2008; Shibley et al., 2008) and to delusions (e.g. Henquet et al., 2008; Morrison et al., 2009; Johns et al., 2004); therefore a mediational role for cannabis use in an association of insomnia and paranoia is also scrutinised. However it is important to highlight that causal relationships cannot be determined in this cross-sectional study.

## 2 Method

The second British National Survey of Psychiatric Morbidity was carried out between March and September 2000. Adults aged 16–74 years and living in private households in Great Britain were sampled. The data used in this study are based on interviews carried out by experienced Office of National Statistics (ONS) interviewers. More details of the topics covered and the methods used are given by Singleton et al., (2001a,b).

### 2.1 Participants

A stratified multi-stage random probability sample was used. There were two stages in the sample selection – the sampling of the primary sampling units (PSUs), followed by the sampling of addresses within the selected PSUs. The PSUs were individual or grouped postcode sectors. In the first stage of sampling, the postcode sectors were stratified on the basis of a measure of socioeconomic status within a regional breakdown. First, postcode sectors were divided into regions based on NHS Regional Office. All the PSUs within each regional stratum were then further stratified on the basis of the proportion of household heads in socioeconomic groups 1–5 and 13, and sorted by the proportion of households without a car based on 1991 census data. The postal sectors (the primary sampling units) were then sampled from each stratum with a probability proportional to size (i.e. the number of delivery points). In this way a total of 438 postal sectors was selected. In the second stage of the sampling, 36 addresses were randomly selected within each of the selected postal sectors. This yielded a total sample of 15,804 addresses. Interviewers visited these to identify private households with at least one person aged 16–74 years. One person was selected from each qualifying household using the Kish (1965) grid method. Just under 70% of those approached agreed to a first phase interview, which the vast majority completed in full (8580: 95%).

### 2.2 Assessments

The current study uses data from three survey instruments: the Clinical Interview Schedule Revised (CIS-R – Lewis et al., 1992), the Psychosis Screening Questionnaire (PSQ; Bebbington and Nayani, 1995), and the self-completion (screening) questionnaire version of the Structured Clinical Interview for DSM-IV (SCID-II; First et al., 1997). The complete list of questions used in the survey is available on the Internet (www.statistics.gov.uk/downloads/theme_health/PMA_2000_AppB.pdf).

Sleep problems were assessed using the CIS-R (Lewis et al., 1992). Insomnia was defined in three ways:

I1. Sleep difficulties: problems in the past month with trying to get to sleep or with getting back to sleep (item D1).
I2. Insomnia of at least moderate severity: problems in the past month with trying to get to sleep or with getting back to sleep, occurring at least four nights in the past week, and taking at least 1 h to attain sleep on an evening in the past week (items D1, D3, D5).
I3. Chronic insomnia: sleep problems for at least six months, problems with trying to get to sleep or with getting back to sleep, occurring at least four nights in the past week, taking at least 1 h to attain sleep on an evening in the past week, together with reports of tiredness (items D1, D3, D5, D10, B1).

Persecutory ideation was also examined in three ways:

P1. Endorsement of the PSQ item ‘In the past year, have there been times when you felt that people were deliberately acting to harm you or your interests?’
P2. Endorsement of the PSQ item ‘In the past year, have there been times you felt that a group of people was plotting to cause you serious harm or injury?’
P3. Total paranoia score. A dimensional measure was constructed by selecting fifteen paranoia-related items from the survey assessments to approximate those of Freeman et al. (2005). We used items 2, 3, 3a and 3b from the PSQ, relating to ideas of persecution, conspiracy and interference. From the SCID-II, we used items 2, 3, 4, 6, 10, 25, 26, 27, 28, 33 and 35 (e.g. ‘Do you spend a lot of time wondering if you can trust your friends or the people you work with?’, ‘When you are out in public and see people talking, do you often feel that they are talking about you?’). The included items were chosen a priori, and progressed in content from mistrust through reference to persecution.

The occurrence of hallucinations was assessed by the PSQ item 5, ‘Over the past year, have there been times when you heard or saw things that other people couldn’t?’

The presence of anxiety, worry, depression and irritability were assessed using the CIS-R (Lewis et al., 1992). For the mediation analysis, computed symptom count scores varying between 0 and 4 were used (DVJ12, DVI11, DVG11, DVE11). A similar computed symptom count (DVD11) was used for insomnia symptoms in this mediation analysis. The questions in the survey on drug use are taken from the U.S. ECA study (Eaton and Kessler, 1985) and self-completed on a computer. In the current study the only drug variable examined was cannabis use in the past year (No/Yes).

### 2.3 Analysis

The main analyses were carried out using the Stata 10.1 survey data analysis commands, as these provide robust confidence limits for the analysis of datasets of complex structure (StataCorp, 2008). Analyses were performed using data weighted to take account of the complex survey design (in particular, the fact only one person per household was sampled) and of non-response, in order to ensure that the results were representative of the British household population aged 16–74 as a whole. Full details of the weighting procedures are available online in the technical report (Singleton et al., 2001b). Logistic and linear regressions were used to test associations of insomnia and paranoia.

The secondary analysis was an examination of the mediation of the relationship between insomnia and paranoia. A model with emotion and cannabis use as potential mediators was assessed using a series of linear and logistic regression models (accounting for the complex survey design). Mediation is a hypothesised causal chain in which an independent variable *X* affects a mediating variable *Y*, which in turn affects the outcome variable *Z* (Baron and Kenny, 1986; MacKinnon, 2008). If the intervening mediator *Y* explains the correlation between *X* and *Z*, we have a *full* mediational model. If *X* still has an effect on *Z* after including the mediator *Y* in the model, the model is consistent with *partial* mediation. The mediation analysis was performed in four steps: i) a linear regression of paranoia scores on insomnia scores was used to estimate the total effect of insomnia ii) the proposed mediators, emotion and cannabis use, were added as independent variables in the first model iii) a linear regression of emotion on insomnia was carried out and iv) a logistic regression of cannabis use on insomnia was performed, which enabled the indirect effects of insomnia on paranoia to be determined. Indirect paths were assessed using the Sobel Aroian Test (MacKinnon et al., 2002). The Sobel tests were produced using a calculator and checked with an online software programme (http://people.ku.edu/∼preacher/sobel/sobel.htm). Because the cannabis use variable was dichotomous, the Sobel Aroian test for this indirect path was modified using the method described by MacKinnon and Dwyer (1993). The results are presented in a path diagram with regression coefficients and 95% confidence intervals. A single emotion variable was created for the mediation analysis by performing a weighted principal axis factor analysis using the four correlated variables depression, worry, anxiety and irritability. The factor analysis was carried out in SPSS (2006) 15.0. It could not be performed using the complex survey design, but the scores derived from separate factor analyses within each area correlated almost perfectly (*r* = 0.99). The meditational regression analyses were also repeated using the four emotion variables individually as possible mediators to examine the size of their effects.

## 3 Results

### 3.1 Presence of insomnia and paranoia

Sleep difficulties were present in 3380 participants (weighted 38.0%), insomnia of at least moderate severity in 1120 participants (weighted 11.9%), and chronic insomnia in 623 participants (weighted 6.6%). The number of individuals endorsing the item ‘Have there been times when you felt that people were deliberately acting to harm you or your interests?’ was 809 (weighted 9.0%). Individuals endorsing the item ‘Have there been times you felt that a group of people was plotting to cause you serious harm or injury?’ numbered 146 (weighted 1.6%). The mean paranoia score was 2.48 (SD = 2.86, minimum = 0, maximum = 14). Information concerning insomnia and paranoia by sex and age is presented in Table 1. Women had higher rates of insomnia, whereas there were few significant sex differences in paranoia. Insomnia increased with age, while paranoia decreased with age. For instance, in the youngest age group (16–25, *n* = 907) 1.9% endorsed the paranoia item P2 and 9.2% reported insomnia (I2), whereas in the oldest age bracket (65–74, *n* = 1268) 0.7% endorsed the paranoia item P2 and 14.1% reported insomnia (all percentages weighted).

### 3.2 Association between insomnia and paranoia

There were strong associations between all the insomnia and paranoia variables (see Table 2). For instance, sleep difficulties in the past month were associated with 2.30 times the likelihood of endorsing the item ‘Have there been times when you felt that people were deliberately acting to harm you or your interests?’. Chronic insomnia increased almost fivefold the likelihood of endorsing the item ‘Have there been times you felt that a group of people was plotting to cause you serious harm or injury?’. The associations became stronger as the severity of insomnia increased, and also as the severity of the content of the paranoid thoughts increased. In linear regressions, the paranoia dimensional score (P3) was significantly associated with sleep difficulties, *b* = 1.41, *p* < 0.001, insomnia, *b* = 1.72, *p* < 0.001, and chronic insomnia, *b* = 2.23, *p* < 0.001. These linear regressions altered little when controlling for age and sex; the paranoia dimensional score (P3) remained significantly associated with sleep difficulties, *b* = 1.44, *p* < 0.001, insomnia, *b* = 1.77, *p* < 0.001, and chronic insomnia, *b* = 2.33, *p* < 0.001. All the associations between sleep difficulties and paranoid ideation remained highly significant (*p* ≤ 0.001) when the analyses were repeated controlling for the presence of hallucinations. Similarly all the associations between sleep difficulties and paranoia ideation remained highly significant (*p* < 0.001) when participants with probable diagnoses of psychosis and participants who had used cannabis in the past year were removed from the analyses.

### 3.3 Explanatory analysis

Symptom counts for paranoia (P3), insomnia, anxiety, worry, depression, and irritability were used for the mediation analysis. Cannabis use in the past year was a dichotomous variable. The paranoia and sleep symptom scores were both positively associated with anxiety, worry, depression, irritability and cannabis use (see Table 3). These associations remained highly significant even controlling for age and sex (*p* ≤ 0.001). A factor score was created for the affective measures. A principal axis factor analysis extracted one factor explaining 44.5% of the variance. All four emotion variables had loadings over 0.5 with the underling factor (Depression = 0.66, Worry = 0.74, Anxiety = 0.68, Irritability = 0.58), justifying the use of factor scores as a response measure of the underlying construct “emotion”.

**Fig. 1:**
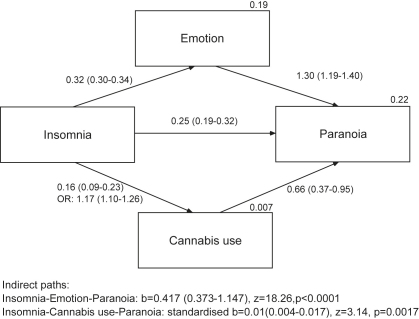
The mediation model. The arrows reflect hypothesised relationships between the variables. Regression coefficients (95% confidence intervals) are shown next to each path. Explained variance of the respective regressions are presented on the top right of the box of the endogenous variables. Nagelkerke’s pseudo *R*^2^ and odds ratios (OR) are presented for the logistic regression of cannabis use on insomnia. Only a standardised effect estimate could be presented for the indirect path with the binary mediator cannabis use.

The result of the mediation analysis is summarized in the path diagram (Fig. 1). All direct paths were highly significant (*p* < 0.0001). A simple linear regression showed that insomnia was positively associated with paranoia, *b* = 0.677, 95% C.I.: 0.611–0.743. The total effect was reduced by 62% to 0.254 when the emotion variable was added into the model. The indirect effect of insomnia via the emotion moderator was 0.417 (0.373–1.147), Sobel Aroian *z* = 18.26, *p* < 0.0001. There was only a small but significant moderating effect of cannabis use, Sobel Aroian *z* = 3.14, *p* = 0.0017; the odds of cannabis use increased by 1.17 for each unit increase of insomnia, and cannabis users scored an average of 0.66 higher on the paranoia scale compared with non-cannabis users. Consequently, adding cannabis use further reduced the direct effect of insomnia to 0.251 (95% C.I.: 0.186–0.315), but this is clearly a very small reduction (1%). Overall, the mediation analysis indicates that the effect of insomnia on paranoia is partially and predominately mediated by the emotion factor score. Using the four emotion variables (anxiety, worry, depression, irritability) in separate regression analysis (including cannabis use) showed that the total effect of insomnia was reduced much less in each case, Worry: *b*_insomnia_ = 0.41 (0.35–0.48), Irritability: *b*_insomnia_ = 0.43 (0.36–0.49), Depression: *b*_insomnia_ = 0.46 (0.39–0.53), Anxiety: *b*_insomnia_ = 0.46 (0.40–0.53). The mediating effect of the emotion variables was consequently smaller than using the factor scores of the latent variable emotion, with worry having the largest effect, then irritability, and anxiety and depression having a similar effect.

## 4 Discussion

Analysis of this large dataset provides substantive replication of the association between insomnia and paranoia. Whether the difficulty in falling or staying asleep was mild or severe, the association with paranoia remained; likewise, whether the paranoid thought was of mild or severe content, the association with insomnia was maintained. The task now turns towards establishing the nature of the association. The most plausible relationship is that sleep difficulties and paranoid fears maintain each other in a circular relationship. However it is also possible that paranoid fears simply lead to withdrawal, inactivity, and rumination that disrupts sleep patterns, or that a third (unidentified) factor explains the association. The most valuable test would be to evaluate the effects of well-established insomnia interventions (e.g. Espie, 2006; Harvey et al., 2007; Freeman and Freeman, 2010) for patients with persecutory delusions and sleep disturbance. This research method has been termed an ‘interventionist causal model approach’ (Woodward, 2003; Kendler and Campbell, 2009).

Putative factors underlying the insomnia and paranoia association were also examined. It was predicted that emotional processes would (partly) explain the relationship. Sleep disturbance produces anxiety, depression and irritability, all factors linked to paranoia; worry also contributes to the occurrence of insomnia (e.g. Nelson and Harvey, 2002) and paranoia (e.g. Startup et al., 2007). In this large dataset the association between insomnia and paranoia was partially, though substantially, accounted for by the affective symptoms. This is consistent with the earlier study (Freeman et al., 2009). However explanatory or meditational relationships are clearly much better tested in longitudinal designs, which are now called for in this area. It also needs to be kept in mind that the overlap between the distinct experiences of paranoia and insomnia is partial, as further evidenced by their opposite relationships to age. In addition to testing an emotional route from insomnia to paranoia, the role of anomalous perceptual experiences requires examination. Sleep deprivation has long been noted to produce temporary psychotic-like experiences (Luby et al., 1960; West et al., 1962), while internal anomalous experiences are considered a key factor in the occurrence of delusions (Maher, 1988; Freeman et al., 2008b). The mediational role found in the current study for cannabis use, which causes anomalies of experience, is supportive of such a route. More broadly, the results of the path analysis are consistent with a burgeoning empirical and theoretical literature indicating a close connection between paranoia and negative affect (e.g. Freeman, 2007; Thewissen et al., 2008; Lincoln et al., 2008). The presence of worry, depression, irritability and anxiety were all strongly associated with paranoid ideation. Paranoid experience has a large affective component. Work translating this knowledge into targeted treatments has already begun (Freeman et al., 2008a; Foster et al., 2010).

## Conflict of interest

None

## Role of funding source

Funding sources had no input to the study described in the article.

## Contributors

Daniel Freeman took the main responsibility for initiating and writing this report of the survey data. Daniel Stahl and Daniel Freeman analysed the data. Traolach Brugha, Howard Meltzer, Rachel Jenkins, and Paul Bebbington were involved in the survey design and commented upon the paper.

## Figures and Tables

**Table 1:** Associations of insomnia and paranoia with sex and age.

Independent variable	Dependent variable	Parameter coding (weighted percentage scoring positively on dependent variable)	Odds ratio or regression coefficient	95% C.I.	*p*-value
Sex	P1	Male (8.8%)		0.90, 1.26	0.442
		Female (9.4%)	OR = 1.07		
	P2	Male (1.7%)		0.53, 1.20	0.278
		Female (1.3%)	OR = 0.80		
	P3	Male		0.21, 0.51	<0.001
		Female	*b* = 0.36		
	I1	Male (35.0%)		1.33, 1.60	<0.001
		Female (44.0%)	OR = 1.46		
	I2	Male (10.8%)		1.19, 1.58	<0.001
		Female (14.2%)	OR = 1.37		
	I3	Male (6.0%)		1.13, 1.63	0.001
		Female (8.0%)	OR = 1.35		

Age	P1	Age	OR = 0.98	0.97, 0.98	<0.001
	P2	Age	OR = 0.98	0.97, 0.99	0.005
	P3	Age	*b* = −0.03	−0.04, −0.03	< 0.001
	I1	Age	OR = 1.01	1.00, 1.01	<0.001
	I2	Age	OR = 1.01	1.01, 1.02	<0.001
	I3	Age	OR = 1.02	1.01, 1.02	<0.001

**Table 2:** Associations of paranoia and insomnia.

	Unadjusted			Age and sex adjusted		
	Odds Ratio	*p*-value	95% C.I.	Odds Ratio	*p*-value	95% C.I.
*P1 dependent variable*: ‘In the past year, have there been times when you felt that people were deliberately acting to harm you or your interests?’						
I1 Sleep difficulties	2.30	< 0.001	1.97, 2.68	2.45	<0.001	2.09, 2.87
I2 Insomnia	2.60	< 0.001	2.17, 3.12	2.83	<0.001	2.35, 3.41
I3 Chronic insomnia	2.67	< 0.001	2.12, 3.37	3.04	<0.001	2.41, 3.84

*P2 dependent variable*: ‘In the past year, have there been times you felt that a group of people was plotting to cause you serious harm or injury?’						
I1 Sleep difficulties	2.33	<0.001	1.55, 3.51	2.48	<0.001	1.65, 3.73
I2 Insomnia	3.33	<0.001	2.20, 5.04	3.58	<0.001	2.37, 5.41
I3 Chronic insomnia	4.50	<0.001	2.91, 6.97	5.03	<0.001	3.24, 7.81

**Table 3:** Associations of paranoia and insomnia with affective symptoms and cannabis use.

	Coefficient (b)	*p*-value	L.C.I.	U.C.I
*Paranoia symptom count (0–15)*				
Anxiety	1.17	<0.001	1.06	1.28
Worry	1.01	<0.001	0.93	1.09
Depression	1.11	<0.001	1.00	1.22
Irritability	1.03	<0.001	0.95	1.11
Cannabis use	1.08	<0.001	0.74	1.41

*Insomnia symptom count (0–4)*				
Anxiety	0.47	<0.001	0.44	0.51
Worry	0.41	<0.001	0.38	0.44
Depression	0.49	<0.001	0.45	0.53
Irritability	0.38	<0.001	0.35	0.40
Cannabis use	0.23	<0.001	0.12	0.34

## References

[bib1] Baron R., Kenny D.A. (1986). The moderator–mediator variable distinction in social psychological research: conceptual, strategic, and statistical considerations. Journal of Personality and Social Psychology.

[bib2] Bebbington P.E., Nayani T. (1995). The psychosis screening questionnaire. International Journal of Methods in Psychiatric Research.

[bib3] Benedetti F., Zanardi R., Colombo C., Smeraldi E. (1999). Worsening of delusional depression after sleep deprivation: case reports. Journal of Psychiatric Research.

[bib50] Bentall R.P., Kinderman P., Moutoussis M., Freeman D., Bentall R., Garety P. (2008). The role of self-esteem in paranoid delusions: the psychology, neurophysiology, and development of persecutory beliefs. Persecutory Delusions.

[bib4] Birchwood M., Smith J., MacMillan J.F., Hogg B., Prasad R., Harvey C. (1989). Predicting relapse in schizophrenia. Psychological Medicine.

[bib5] Breslau N., Roth T., Rosenthal L., Andreski P. (1996). Sleep disturbance and psychiatric disorders: a longitudinal epidemiological study of young adults. Biological Psychiatry.

[bib6] Eaton W.W., Kessler L.G. (1985). Epidemiologic field methods in psychiatry: the NIMH epidemiologic catchment area program.

[bib7] Espie C.A. (2006). Overcoming insomnia and sleep problems.

[bib8] First M.B., Gibbon M., Spitzer R.L., Williams J.B.W., Benjamin L. (1997). Structured clinical interview for DSMIV axis II personality disorders.

[bib9] Ford D.E., Kamerow D.B. (1989). Epidemiologic study of disturbances and psychiatric disorders: an opportunity for prevention?. Journal of the American Medical Association.

[bib10] Foster C., Startup H., Potts L., Freeman D. (2010). A randomised controlled trial of a worry intervention for individuals with persistent persecutory delusions. Journal of Behavior Therapy and Experimental Psychiatry.

[bib11] Freeman D. (2007). Suspicious minds: the psychology of persecutory delusions. Clinical Psychology Review.

[bib13] Freeman D., Freeman J. (2008). Paranoia: the 21st century fear.

[bib12] Freeman D., Freeman J. (2010). Know your mind: the complete family reference guide to emotional health.

[bib14] Freeman D., Freeman J., Garety P. (2008). Overcoming paranoid and suspicious thoughts.

[bib15] Freeman D., Garety P.A., Bebbington P.E., Smith B., Rollinson R., Fowler D. (2005). Psychological investigation of the structure of paranoia in a non-clinical population. British Journal of Psychiatry.

[bib16] Freeman D., Gittins M., Pugh K., Antley A., Slater M., Dunn G. (2008). What makes one person paranoid and another person anxious? The differential prediction of social anxiety and persecutory ideation in an experimental situation. Psychological Medicine.

[bib17] Freeman D., Pugh K., Vorontsova N., Southgate L. (2009). Insomnia and paranoia. Schizophrenia Research.

[bib18] Harvey A.G., Sharpley A.L., Ree M.J., Stinson K., Clark D.M. (2007). An open trial of cognitive therapy for chronic insomnia. Behaviour Research and Therapy.

[bib19] Henquet C., Di Forti M., Murray R.M., van Os J., Freeman D., Bentall R., Garety P. (2008). The role of cannabis in inducing paranoia and psychosis. Persecutory delusions: assessment, theory and treatment.

[bib20] Johns L.C., Cannon M., Singleton N., Murray R.M., Farrell M., Brugha T. (2004). The prevalence and correlates of self-reported psychotic symptoms in the British population. British Journal of Psychiatry.

[bib21] Kendler K.S., Campbell J. (2009). Interventionist causal models in psychiatry. Psychological Medicine.

[bib22] Kish L. (1965). Survey sampling.

[bib23] Lewis G., Pelosi A., Araya R.C., Dunn G. (1992). Measuring psychiatric disorder in the community: a standardised assessment for use by lay interviewers. Psychological Medicine.

[bib24] Lincoln T.M., Peter N., Schäfer M., Moritz S. (2008). Impact of stress on paranoia: an experimental investigation of moderators and mediators. Psychological Medicine.

[bib25] Luby E.D., Frohman C.E., Grisell J.L., Lenzo J.E., Gottlieb J.S. (1960). Sleep deprivation: effects on behaviour, thinking, motor performance, and biological energy transfer systems. Psychosomatic Medicine.

[bib26] MacKinnon D.P. (2008). Introduction to statistical mediation analysis.

[bib27] MacKinnon D.P., Dwyer J.H. (1993). Estimating mediated effects in prevention studies. Evaluation Review.

[bib28] MacKinnon D.P., Lockwood C.M., Hoffman J.M., West S.G., Sheets V. (2002). A comparison of methods to test mediation and other intervening variable effects. Psychological Methods.

[bib29] Maher B.A., Oltmanns T.F., Maher B.A. (1988). Anomalous experience and delusional thinking: the logic of explanations. Delusional beliefs.

[bib30] Morin C.M., LeBlanc M., Daley M., Gregoire J.P., Mérette C. (2006). Epidemiology of insomnia. Sleep Medicine.

[bib31] Morphy H., Dunn K.M., Lewis M., Boardman H.F., Croft P.R. (2007). Epidemiology of insomnia: a longitudinal study in a UK population. Sleep.

[bib32] Morrison P.D., Zois V., McKeown D.A., Lee T.D., Holt D.W., Powell J.F. (2009). The acute effects of synthetic intravenous Δ^9^-tetrahydrocannabinol on psychosis, mood and cognitive functioning. Psychological Medicine.

[bib33] Nelson J., Harvey A.G. (2002). The differential functions of imagery and verbal thought in insomnia. Journal of Abnormal Psychology.

[bib34] Ohayon M.M. (2002). Epidemiology of insomnia: what we know and what we still need to know. Sleep Medicine Reviews.

[bib35] Roth T., Jaeger S., Jin R., Kalsekar A., Stang P.E., Kessler R.C. (2006). Sleep problems, comorbid mental disorders, and role functioning in the national comorbidity survey replication. Biological Psychiatry.

[bib36] Rutten B.P.F., van Os J., Dominguez M., Krabbendam L., Freeman D., Bentall R., Garety P. (2008). Epidemiology and social factors: findings from The Netherlands mental health survey and incidence and incidence study (NEMESIS). Persecutory delusions.

[bib37] Shibley H.L., Malcolm E.J., Veatch L.M. (2008). Adolescents with insomnia and substance abuse: consequences and comorbidities. Journal of Psychiatric Practice.

[bib38] Singleton N., Bumpstead R., O’Brien M., Lee A., Meltzer H. (2001). Psychiatric morbidity among adults living in private households.

[bib39] Singleton N., Lee A., Meltzer H. (2001). Psychiatric morbidity among adults living in private households. http://www.statistics.gov.uk.

[bib40] SPSS (2006). SPSS Base 15.0 User’s Guide.

[bib41] Startup H., Freeman D., Garety P.A. (2007). Persecutory delusions and catastrophic worry in psychosis: developing the understanding of delusion distress and persistence. Behaviour Research and Therapy.

[bib42] StataCorp (2008). Stata statistical software: release 10.0.

[bib43] Stein M.B., Belik S.-L., Jacobi F., Sareen J. (2008). Impairment associated with sleep problems in the community: relationship to physical and mental comorbidity. Psychosomatic Medicine.

[bib44] Stewart R., Besset A., Bebbington P., Brugha T., Lindesay J., Jenkins R. (2006). Insomnia comorbidity, impact and hypnotic use by age group in a national survey population aged 16–74 years. Sleep.

[bib45] Thewissen V., Bentall R.P., Lecomte T., van Os J., Myin-Germeys I. (2008). Fluctuations in self-esteem and paranoia in the context of daily life. Journal of Abnormal Psychology.

[bib46] Walsh J.K. (2004). Clinical and socioeconomic correlates of insomnia. Journal of Clinical Psychiatry.

[bib47] West L.J., Janszen H.H., Lester B.K., Cornelisoon F.S. (1962). The psychosis of sleep deprivation. Annals of New York Academy of Sciences.

[bib48] Woodward J. (2003). Making things happen: a theory of causal explanation.

[bib49] Yung A.R., McGorry P.D. (1996). The prodromal phase of first-episode psychosis. Schizophrenia Bulletin.

